# A systematic review of entomological outcomes and sampling approaches used in the evaluation of cluster randomised controlled trials for malaria vector control products

**DOI:** 10.1186/s12936-026-05866-4

**Published:** 2026-03-22

**Authors:** Victoria Githu, Praise Michael, Joseph Biggs, Jackie Cook, Samson Kiware, Alex McConnachie, Paul Johnson, Heather M. Ferguson

**Affiliations:** 1 School of Biodiversity, One Health and Veterinary Medicine, University of Glasgow, Glasgow, UK; 2 School of Health and Wellbeing, Robertson Centre for Biostatistics, University of Glasgow, Glasgow, UK; 3 Environmental Health, and Ecological Sciences Department, Ifakara Health Institute, Kiko Avenue, Mikocheni, Dar es Salaam, United Republic of Tanzania; 4 International Statistics and Epidemiology Group, Department of Infectious Disease Epidemiology and International Health, London School of Hygiene and Tropical Medicine, London, UK

**Keywords:** Malaria vector control, Entomological outcomes, Cluster randomised controlled trials

## Abstract

**Background:**

Entomological outcomes are critical for understanding the biological mechanisms and operational performance of malaria vector control interventions. However, the quality and consistency of how these outcomes are measured and reported in cluster randomised controlled trials (cRCTs) is unclear. We conducted a systematic review of design features, sampling methods, and metrics used for measuring entomological outcomes in malaria vector control cRCTs. Our aims were to assess the consistency and quality of entomological study designs and examine how key design features influence the precision of reported entomological effect sizes.

**Methods:**

Malaria cRCTs in the published literature were identified in four databases and two trial registries. We extracted information on entomological outcomes measured, entomological sampling strategies and trial design features. To evaluate overall design quality, we created an Entomological Study Design Risk (ESDR) metric that assessed trials across four domains related to power analysis and sampling design; with domain ratings combined to generate an overall ESDR score. We also used a linear mixed-effects model (LMM) to examine how the use of power analysis and the number of clusters and sampling points in entomological sampling influenced the precision of main entomological outcomes.

**Results:**

Sixty-two cRCTs of malaria vector control interventions were identified between 1992 and 2021; with 74% being conducted in Africa. Thirteen different entomological outcomes and 12 sampling methods were used across trials: with considerable variation in the frequency of entomological sampling within trials. In total, 70% of the cRCTs were categorized as having high potential entomological design risk based on a combination of the lack of power analyses and inadequate or unclear randomisation of sampling points. The only significant predictor of the precision of entomological outcomes was the number of entomological clusters in which data were collected (greater precision as cluster number increased).

**Conclusions:**

There is substantial variation in how entomological outcomes are measured and reported in cRCTs, and limited incorporation of key design features such as power analysis and random selection of sampling points for outcome measurement. Standardization where possible and clearer reporting guidelines for entomological components are therefore needed to improve the quality, comparability, and policy relevance for future trials.

**Supplementary Information:**

The online version contains supplementary material available at 10.1186/s12936-026-05866-4.

## Background

Vector-borne diseases (VBDs) remain a major global health concern, with malaria alone causing an estimated 282 million cases in 2024 [1, 2]. Although the first malaria vaccine has recently been rolled out in 19 African countries [3], mosquito vector control remains central to malaria prevention [4]. Core interventions recommended by the World Health Organization (WHO) include indoor residual spraying (IRS) and insecticide-treated nets (ITNs); with larval source management (LSM) recommended as a supplementary approach in suitable settings [5]. However, challenges, such as insecticide resistance and inadequate coverage, threaten their impact [6, 7]; with modelling indicating that even high coverage of current frontline interventions will not achieve elimination in high-transmission areas [8]. This underscores the need for continued research and investment in novel and more effective vector control strategies [4].

The development of policies on the use of vector control interventions to combat malaria depends on high-quality evidence of their impact. While evidence on vector control effectiveness can be obtained from a range of study designs, cluster randomised controlled trials (cRCTs) are widely regarded as the gold standard [9]. As vector control interventions often provide protection at the community rather than just individual level [10], they are often assessed through comparison of disease incidence or prevalence between clusters in different arms; with cluster referring to a geographical unit such as a district, village, community, or block (sum of households) [10]. Entomological impacts of vector control interventions arise through their impact on the mosquito vector population and its transmission capacity, which are measured through collection of entomological outcomes such as mosquito density, human biting rates, malaria infection rates in mosquitoes, and the composite outcome of the ‘entomological inoculation rate’ (EIR, defined as the mean number of infectious bites a person would be expected to receive per unit of time [11]). Notable reductions in any of these variables would be expected to lead to decreased human exposure to infection. In contrast, epidemiological outcomes pertain to direct health impacts on human populations, including measures such as infection and/or disease incidence and prevalence, morbidity, and mortality rates [12]. Vector control interventions can be assessed based on entomological outcomes alone, epidemiological outcomes alone, or both. The WHO prioritizes measurement of epidemiological outcomes as required to assess the direct public health value of new vector control tools, but also recommends collection of robust entomological data within vector control trials to contextualize findings [13]. The quality and rigour with which both types of outcomes are collected determines their value for assessing intervention impact.

When assessing the value, rigour and generalizability of findings based on entomological outcomes, there are two important considerations: (1) the selection of entomological outcomes and methods used to record them (sampling methods) and (2) the rigour of how entomological measurements is carried out. The methodologies employed for entomological surveillance can exhibit significant variability [14, 15]. This methodological heterogeneity can be attributed to a number of factors such as funding and logistic constraints, differences in the specific mode of action of the intervention (e.g., whether targeting adult or larval mosquitoes, their transmission potential, or resistance profiles), local ethical regulations (e.g., the permissibility of human landing catches in certain regions), and the availability of appropriate sampling tools and expertise [16]. We highlight that the use of different entomological sampling methods across trials is not necessarily problematic. There is often a solid rationale for choosing different sampling methodologies depending on the ecological context and behaviors of primary vector species (e.g. indoor, human baited trapping approaches where vectors are anthropophagic and endophilic; vs outdoor, animal-baited traps when they are more zoophagic and exophilic). In such circumstances, enforcing a standard ‘one size fits all’ sampling would be inappropriate; and fail to capture impacts relevant to different ecological and intervention contexts. While it is reasonable to adapt entomological sampling methods to local contexts and study aims, lack of standardization where possible (ie. for similar vector species and intervention types) can make it difficult to interpret and compare results across studies.

Another important source of variation in vector control trials is the choice of design features to measure entomological outcomes. Well-designed cRCTs should incorporate several key features to ensure the reliability and interpretability of findings; such as the use of pre-trial data to enhance balance between arms, random allocation of control and intervention clusters, standardized measurement of outcomes, and unbiased selection of subjects or sampling points for data collection [10]. Crucially, robust sample size calculations are essential to determine the appropriate number of sampling units (clusters, individuals/household sampling points) required to detect the epidemiological or entomological effect sizes of interest [10, 17]. While conducting sample size calculations to guide the measurement of epidemiological outcomes is often a requirement for obtaining funding and ethical approval for cRCTs, there may be less rigour and attention given to powering entomological outcomes. Pre-trial data is crucial for robust power analysis, as this enables estimation of heterogeneity between clusters and balancing of baseline characteristics between arms. Another discrepancy is the spatial coverage and intensity with which entomological and epidemiological outcomes are measured in cRCTs. While epidemiological outcomes are typically collected in all study clusters, entomological sampling may be deprioritized and limited to only a subset of clusters due to resource constraints. The way in which the subset of clusters for entomological sampling is selected could bias estimation of intervention impact; especially if based on non-random selection. Additionally, non-random selection of entomological sampling units (typically households) could introduce bias and limit generalisability. Finally, the temporal and spatial independence of entomological sampling points in cRCTs could also impact the accuracy and precision of entomological outcomes. For example, the same households (often defined as ‘sentinel households’) are repeatedly used for entomological sampling in some cRCTs, potentially leading to high precision but lower spatial coverage of sampling, whereas other cRCTs will select a different group of households for entomological sampling at each data collection period. Both approaches could be justifiable, however non-random selection of household sampling points with either approach could create bias.

High variability in the methodology and quality of design features used to measure entomological outcomes could explain differences in the estimated impact of vector control interventions in different trials and impede comparison between studies, posing a challenge for policy decision-making where recommendations for vector control tools rely on pooled evidence from multiple trials. However, the extent of variability in the choice and quality of design features used to measure entomological outcomes is unclear, with limited understanding of whether certain methodological or design choices lead to more accurate or precise effect size estimates. To assess the nature of this problem and identify areas for improvement, we conducted a systematic review of the design features and methodological approaches used to assess entomological outcomes in cRCTs of malaria vector control interventions. Key objectives were to (1) characterize how entomological outcomes are typically measured in terms of outcome choice, the frequency, geographic and temporal distribution of sampling points across the study area, and sampling methods used, (2) assess the quality of entomological design features using a novel Entomological Study Design Risk (ESDR) framework that summarises the approach to entomological outcome measurement across four core domains: use of power analysis, incorporation of clustering in sample size calculations for entomology, randomisation of clusters for entomological assessment, and randomisation of household sampling points, and (3) test whether the precision with which entomological outcomes are estimated in cRCTs is associated with design features. Addressing these objectives will help strengthen guidance on the design, implementation, and reporting of entomological components in future vector control cRCTs, with the ultimate goal of supporting more accurate interpretation of intervention effects on mosquito vector populations.

## Methods

This systematic review was conducted following the principles of Preferred Reporting Items for Systematic Reviews and Meta-Analyses (PRISMA) [18] with a protocol published in PROSPERO [19].

### Literature retrieval and selection criteria

We searched systematically for cRCTs of vector control interventions for malaria in English language, with status limited to published or in press. The search was conducted on 16th August 2024 on MEDLINE, Embase, Web of Science and Global Index Medicus databases together with ClinicalTrials.gov and CENTRAL (The Cochrane Central Register of Controlled Trials) registries. The search strategy consisted of a combination of truncated versions of ‘malaria’, ‘cluster randomised trial’, and ‘vector control products’ (see Supplementary Information 1 for the full search strategy and results). All studies obtained through the initial search were entered into EndNote 21.5 (Clarivate) to review and remove duplicates. After screening titles and abstracts, studies were selected for data extraction by meeting the inclusion criteria of (1) malaria being the targeted disease, (2) assessment of a vector control intervention, and (3) being a cluster randomised trial with a minimum of 3 clusters randomized to an intervention arm.

### Data extraction

We extracted data encompassing details of trial settings (where the trial was conducted and its characteristics), intervention type and trial duration, and aspects of entomological sampling methods and study designs that were aligned with our objectives. This included extraction of the type of outcomes collected (i.e. entomological only, epidemiological only or both), the choice of entomological outcomes reported, and the sampling methods employed (e.g., trap types, frequency and timing of sampling). To assess historical trends in the measurement of entomological outcomes, we extracted publication years, trial start and end dates, and study locations. To examine the study designs used to capture entomological outcomes, we extracted data on trial features including randomization design (matched, unmatched, restricted or stratified), whether clusters and household sampling points for entomological assessment were randomly selected, and whether power analyses were performed for entomological outcomes. To quantify how design features influenced the precision of entomological effect estimates, we extracted effect size values for main entomological outcomes together with their associated confidence intervals and p-values. We also summarized the statistical analysis methods used for entomological outcomes in trials, and qualitatively reviewed the analysis, assessing whether they were appropriate and likely to generate unbiased confidence intervals, particularly in relation to accounting for clustering.

### Data analysis

Data analysis was conducted using the R statistical software version 4.4.2 [20] with all visualizations created using the ggplot2 package [21]. To address the first objective, we generated a map of the geographic distribution of all identified vector control trials. Trends in entomological sampling methods, entomological outcomes and temporal trends in vector control trials were summarized descriptively.

To address the second objective, we examined key trial design features related to the collection of entomological data. The first three criteria (i) use of pre-trial data (entomological only, epidemiological only or both outcomes) to inform allocation of study arms, (ii) choice of clusters for entomological versus epidemiological outcomes in trials measuring both, and (iii) location and frequency of sampling were characterized descriptively. These features reflect variation in practice but, to date, there is no clear evidence or consensus on which specific approaches are superior, nor established recommendations that favour one choice over another. In contrast, the second set of criteria; (iv) whether a power analysis was used to guide entomological sampling and whether it accounted for clustering, and (v) whether clusters and household sampling points were randomly selected are supported by explicit guidance from WHO [13] and by recommendations from previous methodological reviews [10, 15] as essential components of rigorous vector control trial design. As these features are directly linked to reducing bias and improving study validity, we incorporated them into a structured assessment through development of a novel *Entomological Study Design Risk* (ESDR) metric informed by the Cochrane Risk of Bias framework [22] but modified to reflect entomology-specific domains.

The ESDR evaluated four domains: D1—whether a power analysis was reported for entomological outcomes; D2—whether clustering was accounted for in the entomological power analysis; D3—whether clusters were randomly selected for entomological outcome measurement; and D4—whether household sampling points were randomly selected for entomological outcome measurement. Each domain was ranked using a three-level scale: low risk (green), some concerns (yellow), and high risk (red); details on how each domain was ranked are provided in Supplementary file 2. Each cRCT that reported entomological outcomes was assessed independently across the four domains. An overall ESDR score was then assigned to each trial using a risk of bias approach: trials were classified as high risk if they had a high-risk rating in any domain; as some concerns if they had no high-risk ratings but at least one domain rated as ‘some concerns’; and classified as low risk only if all four domains were rated as low risk. Domain-level ratings were summarised by calculating the percentage of trials falling into each risk category for each domain.

To address the third objective, we fitted a Gaussian linear mixed-effects model (LMM) using the lme4 package [23] to estimate associations between the precision of the intervention effect estimate and three trial design features: whether a power analysis for entomological outcomes was done (yes/no), the number of clusters where entomological data was measured per arm and the number of unique household sampling points for entomology per cluster. As multiple entomology outcome results could be reported in the same study (e.g. for different vector species or different time points), a random effect of study ID was included in the model to account for between-study heterogeneity. Log-transformed intervention estimate precision was used as the outcome variable of the LMM. The precision of an estimate is defined as the inverse of its variance, therefore in this analysis, precision was calculated as the inverse of the squared standard error. We assumed that confidence limits around rate and odds ratios were calculated as exp(effect estimate ± 1.96 × standard error), thus the standard error was estimated as the difference between the natural log confidence interval bounds, divided by 3.92, which is the width of the confidence interval in standard error units. Finally, precision was natural-log-transformed to meet the assumptions of normal distribution of residuals and random effects. As the effect sizes analysed are expressed as ratios, they and their confidence intervals are scale-free and therefore comparable across studies. Prior to analysis, we reviewed whether the statistical analysis of each trial appropriately accounted for clustering, as this is necessary to produce unbiased standard errors and confidence intervals in cRCTs.

Univariable and multivariable LMM models were fitted, with a significant association defined as p < 0.05 using likelihood ratio tests. Although individual predictors were not significant in univariable analyses, the number of entomological clusters remained significant in the multivariable model. The effect sizes themselves were not used directly in the subsequent analyses, as they should not be interpreted as an indication of the quality of design features (e.g. an effect size of zero is not a weakness if the intervention was truly ineffective; and a large effect size may not be interpretable, for example if it has a very wide confidence interval that includes zero). Our focus on the precision of entomological effect sizes, rather than their values, provides a direct assessment of statistical power. Effect size estimate precision (and its close relative, confidence interval width) is the best gauge of the power achieved by a trial post hoc [24]. A risk-of-bias assessment was also conducted for the trials included in the LMM as a basic appraisal of study quality, using an adaptation of the Cochrane Risk of Bias tool [22] for entomological outcomes. Detailed assessments of Cochrane risk of bias for the subset of studies are presented in supplementary file 2.

## Results

### Overview of the included studies

**Fig. 1:**
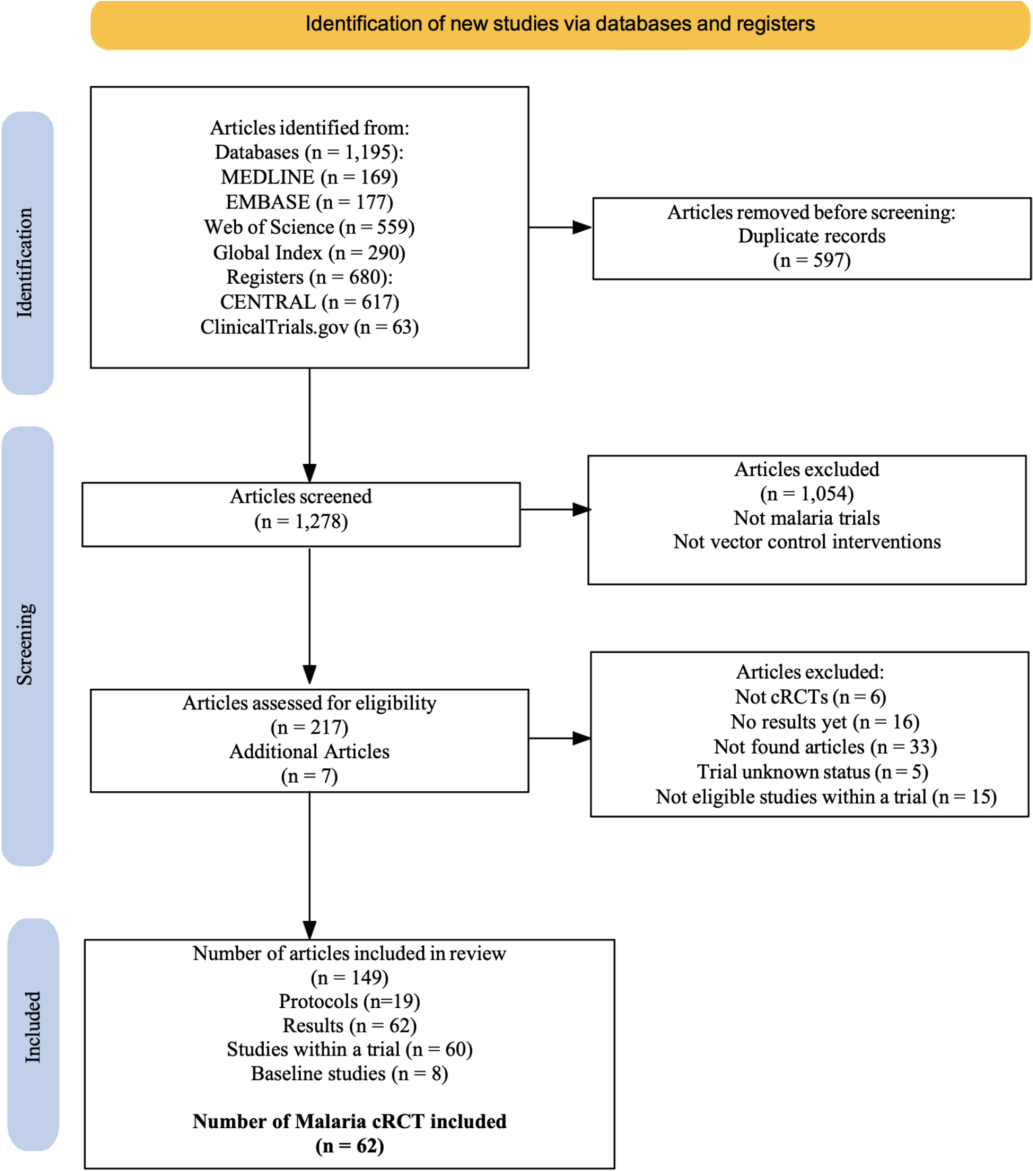
PRISMA diagram describing the total number of articles identified on malaria vector control cRCTs via literature searches, how these were classified during screening, and the total number that were retained as meeting the inclusion criteria for this study of having required information of measuring epidemiological and/or entomological impact

Among the 1875 articles initially retrieved from the four databases and two registries, 597 duplicates were identified. A total of 1278 articles were screened of which 1058 were excluded for not being malaria cRCTs and/or vector control interventions. The remaining 217 articles were supplemented by an additional seven articles that were identified through screening. All 224 articles were assessed for eligibility by full text screening: resulting in the final inclusion of 149 articles in this review. These articles consisted of 62 malaria vector control cRCTs from 1992 to 2021: including 19 protocols, 8 baseline studies, 62 main study results and 60 secondary results records (Fig. 1). Forty six of the 62 trials (64%) reported entomological outcomes [25–70].

### Inclusion and choice of entomological outcomes

**Fig. 2:**
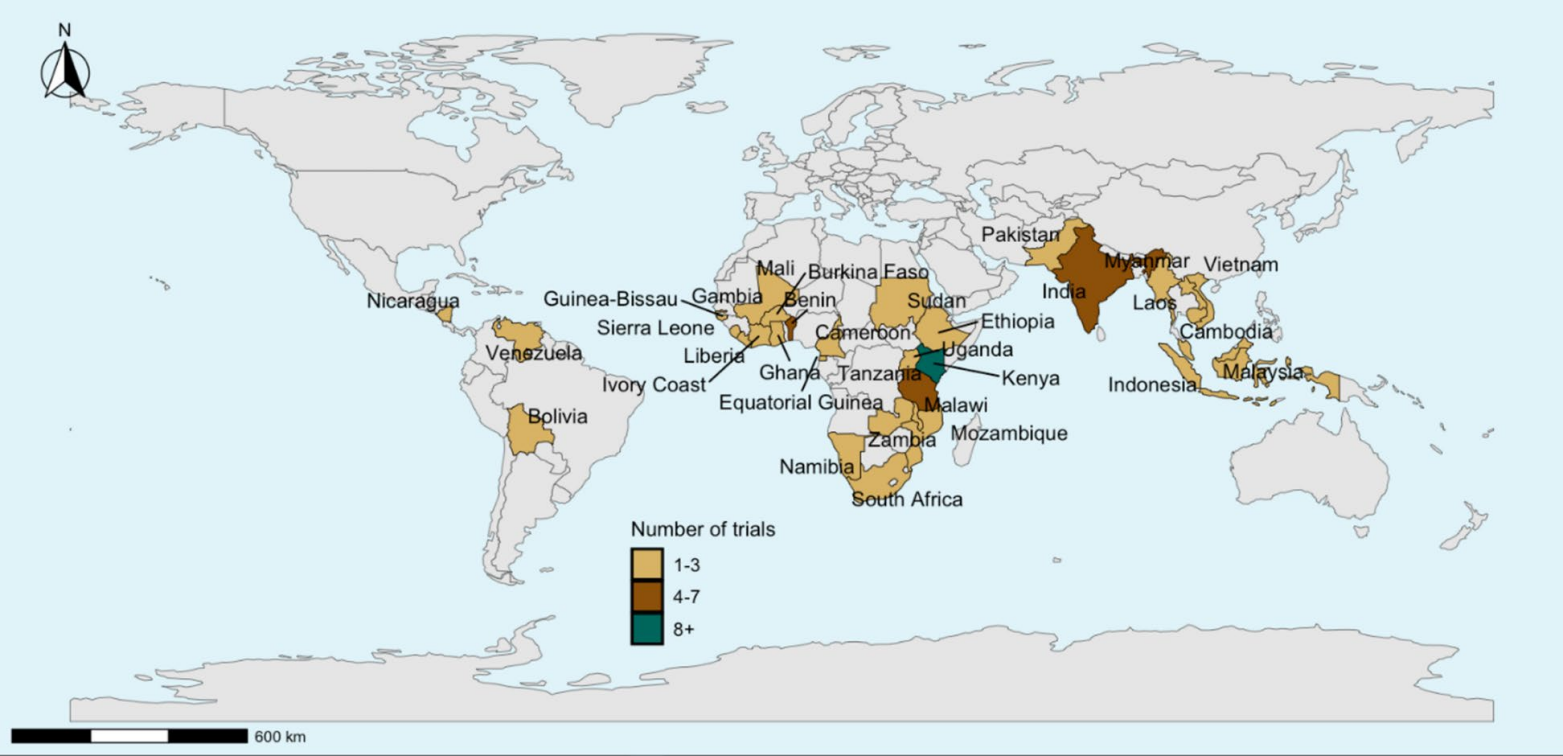
The geographical distribution of the malaria vector control cRCTs across 32 countries detailing the number of trials conducted in each country

**Fig. 3:**
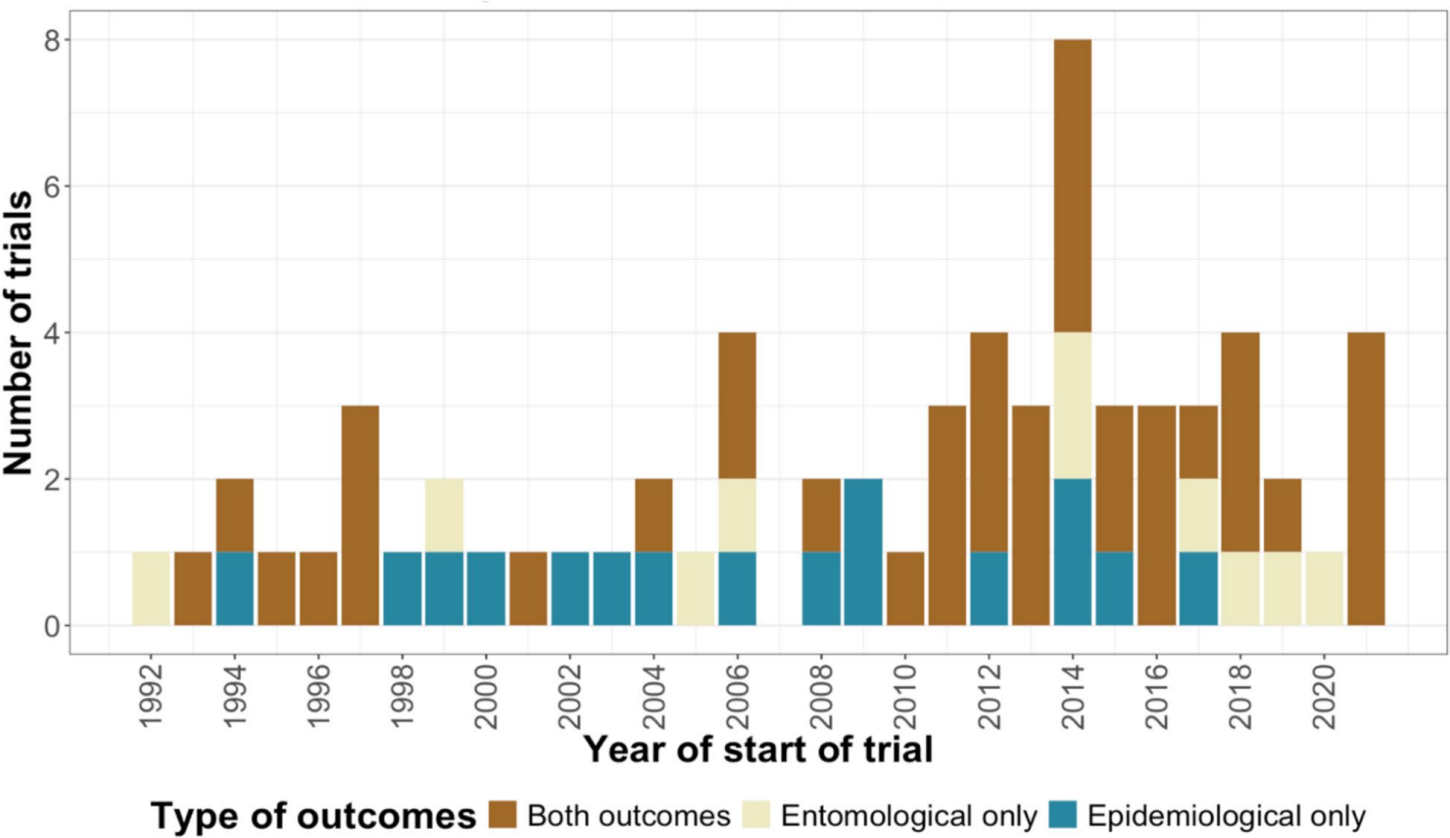
Temporal progression of malaria vector control cRCTs categorized by the type of outcomes assessed (epidemiological, entomological, or both entomological and epidemiological)

The 62 malaria vector control cRCTs identified were distributed in 32 countries (Fig. 2), with most (74%) conducted in Africa. The geographical distribution of trials was notably uneven, with a marked concentration in East Africa (Kenya, Tanzania, Ethiopia, and Uganda); which together accounted for nearly half of all African trials. Between 1992 and 2021, there was an increasing trend of vector control cRCTs with a noticeable rise between 2012 and 2016 (Fig. 3). Sixty-five percent of these trials had an unmatched randomization design, with a similar percentage assessing only one intervention relative to no intervention or a standard of care control arm (i.e. two study arms, Table 1). Sixteen percent of trials (10/62) collected entomological outcomes only, with the majority (36/62, 58%) assessing both entomological and epidemiological outcomes. Of trials that collected both outcomes, only 35% (16/36) had entomological outcomes as the primary outcome.

**Table 1:** Main entomological outcomes, sampling methods and types of interventions assessed in malaria cRCTs

Type	Variable	Trials	Percentage
Named main entomological outcome (trials with entomological outcomes N = 46)	Mosquito density	26	56
	Human Biting Rate (HBR)	6	13
	Entomological Inoculation rate (EIR)	6	13
	Insecticide resistance (IR)	3	7
	Parity	3	7
	Bio-efficacy	2	4
Sampling methods used (trials with entomological outcomes N = 46)	Centre for Disease Control (CDC) Light trap	17	38
	Human Landing Catch (HLC)	12	27
	Pyrethrum Spray Catch (PSC)	6	14
	Exit traps	3	5
	Larvae collection	2	4
	Cow Baited Trap (CBT)	1	2
	Prokopack aspirators (PA)	1	2
	Space Spray Catches (SSC)	1	2
	Suction tube collections	1	2
	Suna traps	1	2
	Not mentioned	1	2
Location of sampling	Indoor	20	43
	Outdoor	3	7
	Both	22	48
	Not described	1	2
Time points for entomological follow up	Nightly	1	2
	Weekly	3	7
	Biweekly	7	16
	Monthly	20	43
	Bimonthly	5	11
	Every 6 weeks	1	2
	Quarterly	2	4
	Round (3 monthly interval & after 10 months—Mass drug administration rounds)	1	2
	No schedule (Larvae collection)	2	4
	Not stated	4	9
Vector control interventions assessed (all trials N = 62)	Insecticide Treated Nets (ITN)	20	32
	Indoor Residual Spraying (IRS)	12	19
	Spatial Emanator (SE)	7	11
	Larvae Source Management (LSM)	3	5
	Endectocides	4	6
	ITN & IRS	4	6
	House Modification (HM)	1	2
	Insecticide Treated Materials (ITM)	3	4
	Attractive Targeted Baits (ATB)	2	3
	ITN & ITM & IRS	1	2
	ITN & LSM	1	2
	LSM & HM	1	2
	Lethal house lure	1	2
	Outdoor Residual Spraying (ORS)	1	2
	Wall lining	1	2

A range of entomological outcomes were measured in the trials, including mosquito density, sporozoite rate, entomological inoculation rate (EIR), human biting rate (HBR), species composition, insecticide resistance status, parity, human blood index, fecundity, and larval density in aquatic habitats. The main epidemiological outcomes were consistently clearly stated in these cRCTs, while when multiple entomological outcomes were measured, only 59% (27/46) of trials clearly designated of which ones were considered the main or additional entomological outcome(s). Of these, adult mosquito density was most commonly designated as the main entomological outcome (56%, 26/46 trials). Other commonly reported main entomological outcomes were HBR, EIR, parity and bio-efficacy (Table 1).

A range of mosquito sampling methods were used to collect data on entomological outcomes (Table 1). The most frequently used method was CDC light traps (used indoors in 9/17 trials, and indoors and outdoors in 8/17 trials). It was also common for cRCTs to use multiple mosquito collection methods, as required to estimate different types of entomological outcomes. For example, 54% of trials used a single entomological collection method, 28% used two methods, and the remainder used three to four. Some trials also reported collecting mosquito larvae from aquatic habitats and rearing them to adulthood in an insectary/laboratory to assess bio-efficacy and monitor insecticide resistance. With the exception of trials that included larval surveys, most entomological sampling points were defined as households within clusters. Sampling of entomological outcomes at households occurred only indoors in 43% of cRCTs (20/46, Table 1), both indoors and outdoors in 48%, with the remainder being outdoor only (Table 1). There was variability in the frequency of entomological outcome measurement within trials. At least nine different sampling schedules were used, with monthly sampling being the most common (43%, 20/46). In addition to the frequency of sampling, the number of nights per sampling period also varied considerably across studies. Many trials (50%) relied on collections from a single night per sampling point, while 20% and 11% reported conducting mosquito sampling over two or three/four nights respectively. A smaller proportion extended sampling to five/more nights or did not report the number of sampling nights per time point.

**Fig. 4:**
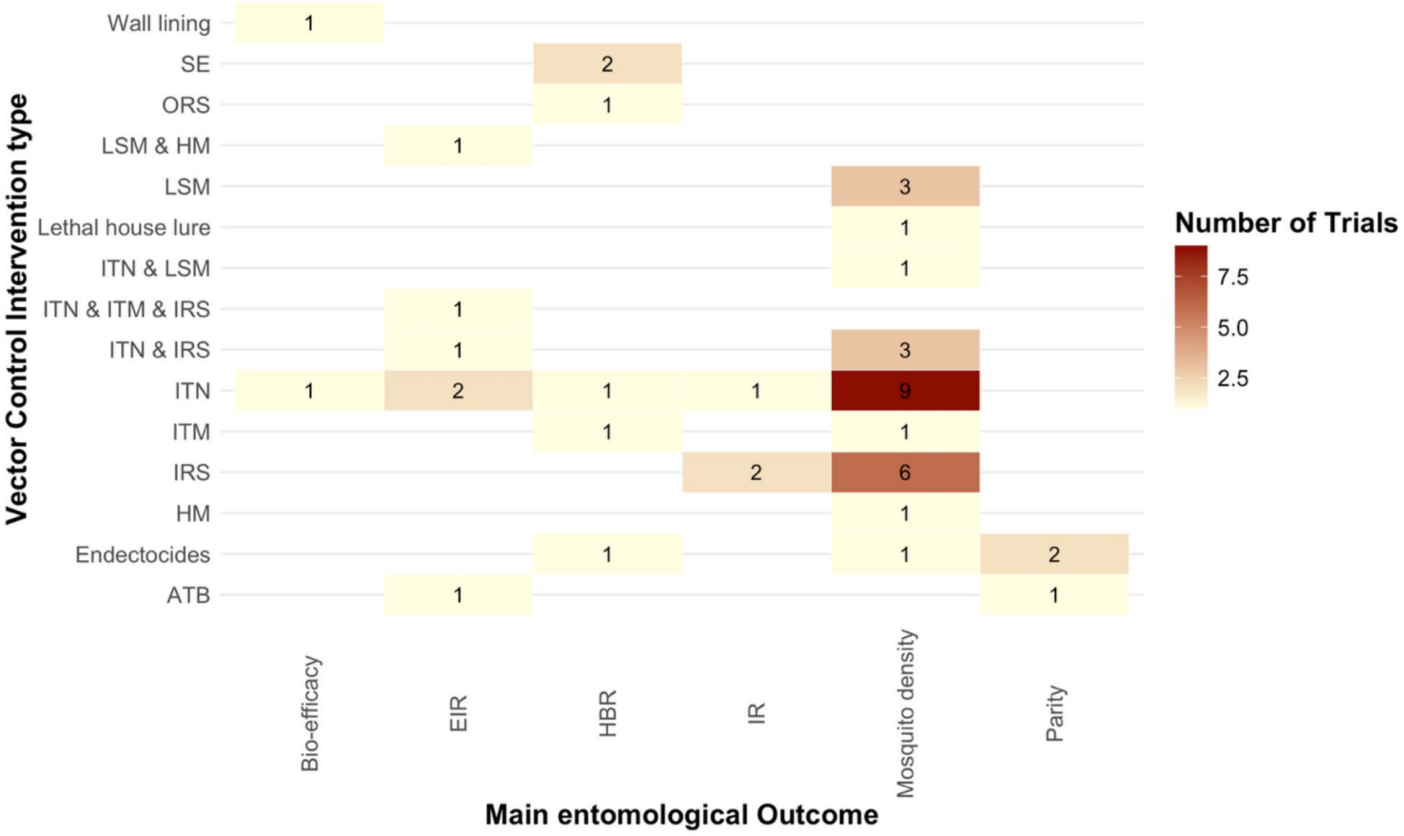
Heatmap showing the distribution of main entomological outcomes measured across vector control interventions in the cRCTs included. Cell values represent the number of trials using each intervention vs outcome combination

A range of intervention types and products were evaluated in the selected vector control cRCTs (Table 1), with ITNs and IRS being the most common. Most other intervention types were assessed in 4 or less cRCTs, with the exception of spatial emanator (SE, 7 cRCTs). The distribution of entomological outcomes measured across these intervention types is shown in Fig. 4, which highlights variability not only across intervention categories but also within individual intervention types, where multiple outcomes were used to evaluate similar interventions.

### Study design qualities and methodological features

**Fig. 5:**
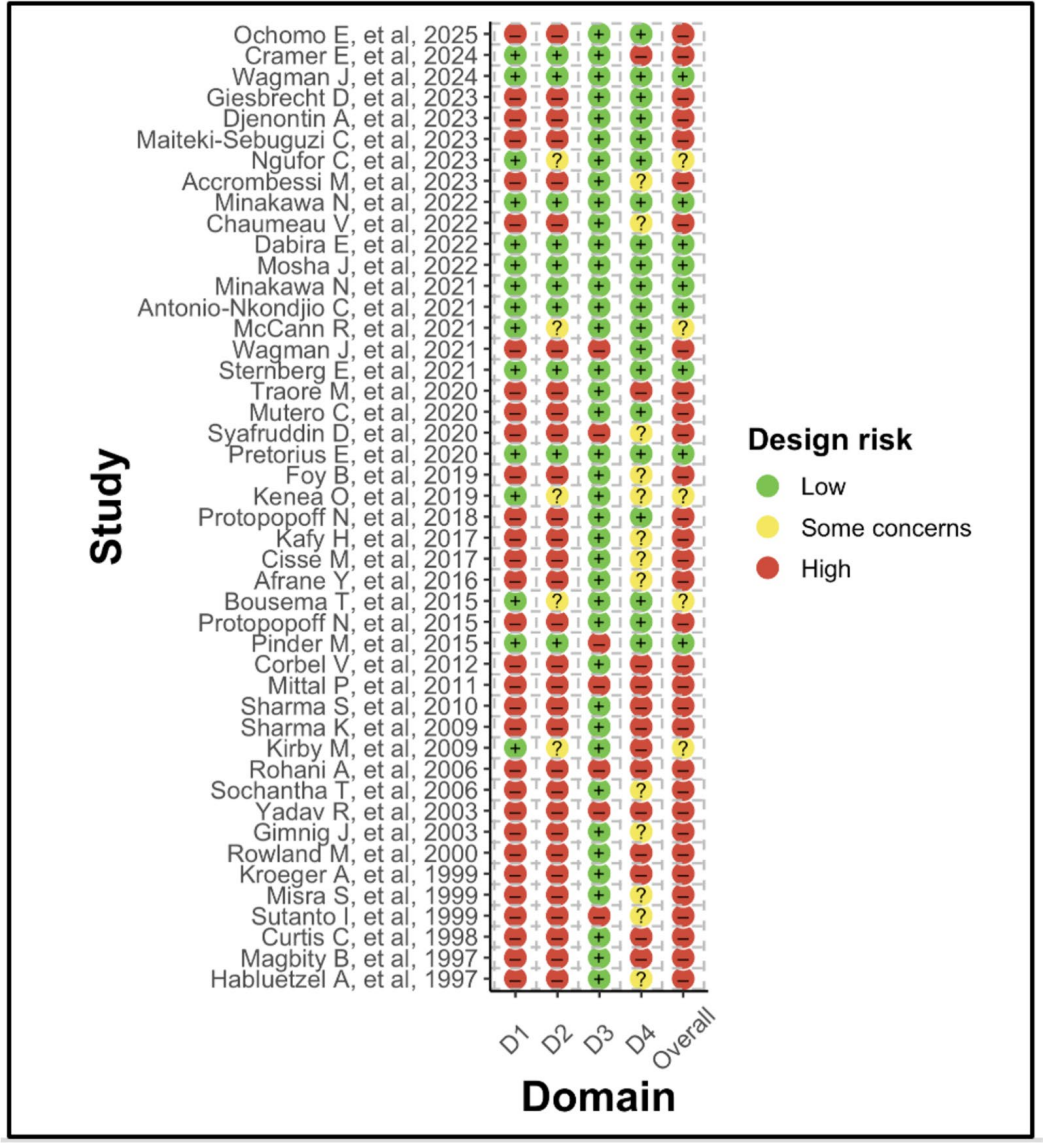
Entomological Study Design risk framework for trial design features assessment: individual study judgements across domains. D1: whether a power analysis was reported for entomological outcomes; D2: whether clustering was accounted for in the entomological power analysis; D3: whether clusters were randomly selected for entomological outcome measurement; and D4: whether household sampling points were randomly selected for entomological outcome measurement

**Fig. 6:**
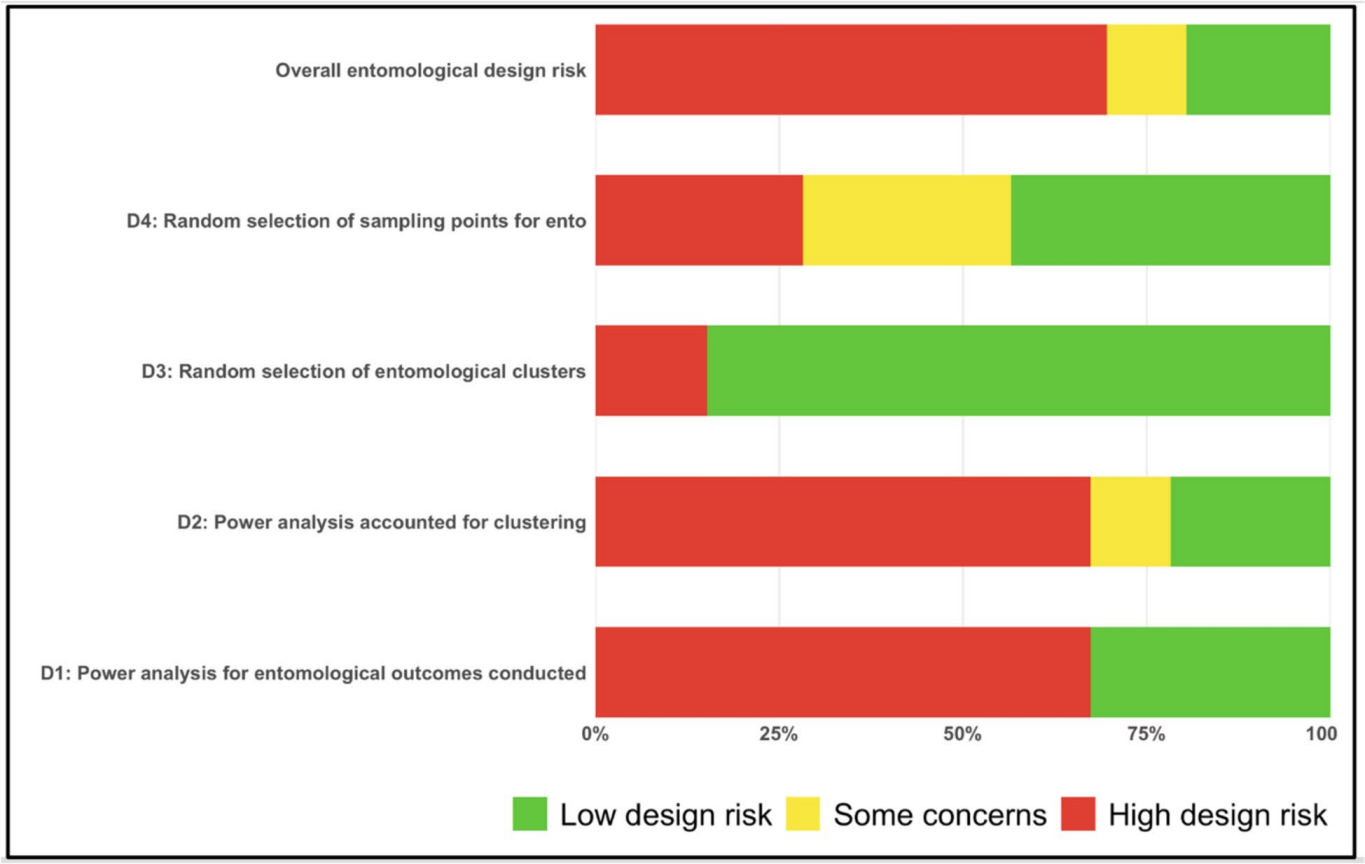
Entomological Study Design risk framework for trial design features assessment: Aggregated percentages across domains

Thirty percent (14/46) of trials collected pre-trial data (epidemiological and/or entomological) from the trial site to guide sample size calculations (Table 2), The other 70% of cRCTs did not use their own pre-trial data but used data (epidemiological or entomological or both) from studies conducted previously in or close to the same area (e.g. similar districts/region) as the trial. While almost all trials with epidemiological outcomes conducted sample size calculations to determine the number of clusters and sampling points for these outcomes, most cRCTs did not report any power analyses for entomological outcomes (ESDR framework, D1, 31/46, 67%). Similarly, in cRCTs that did report sample size calculations for entomology, only 60% accounted for clustering in their power analyses (D2, 9/15). By contrast, most studies reported random selection of clusters for entomological assessment (D3 Fig. 5), with this design choice considered low risk. For trials that collected epidemiological and entomological outcomes, 61% (22/36) measured both in all study clusters, with entomological measurements being restricted to a subset of epidemiology clusters in the remainder (Table 2). The selection of household sampling points for entomological data (D4) contributed substantially to high design risk across trials. Thirteen of the 46 trials (28%) were categorised as high risk because they did not randomly select households for entomological sampling and instead reported relying on convenience-based or proximity-based approaches. A further 13 trials (28%) were rated as having some concerns, either because the method of household selection was unclear or because only partial randomisation was used (e.g., selecting the first household at random and choosing subsequent households non-randomly). When aggregated across all four domains of the ESDR framework, the majority of cRCTs (70%) were classified as having a high entomological study design risk, most commonly driven by the lack of power analyses combined with no or inadequate applied household randomisation (Fig. 6).

**Table 2:** Frequencies and percentages of study design properties for trials with entomological outcomes

Type	Variable	Trials	Percentage
Overall randomisation design of a trial	Unmatched	21	46
	Restricted	11	24
	Matched	7	15
	Stratified	7	15
Number of study arms	2	30	65
	3	8	17
	4	8	17
Use of pre-trial data for allocation of clusters in a trial	No	32	70
	Entomological data only	5	11
	Epidemiological data only	4	8
	Both outcome data	5	11
Which clusters were used for entomological data collection? (From trials with both outcomes N = 36)	Same as epidemiological clusters	22	61
	Randomly selected from epidemiological clusters	8	22
	Chosen out of easy access/high mosquito abundance/budget constraints	6	17
Sampling points at entomological data collection time point	Different sampling points	16	35
	The same sampling points	23	50
	Not stated	7	15

## Predictors of entomological precision

Of the 46 trials that reported entomological outcomes, only 25 provided sufficiently detailed and extractable entomological effect size with corresponding uncertainty estimate results for calculation of precision. Trials conducted in more recent years tended to report results more clearly and comprehensively, with all cRCTs included in this precision analysis being published after 2009. All 25 trials from which precision estimates were derived explicitly accounted for clustering in their analyses, though the strategies used varied (ranging from incorporation of random effects at the cluster level, robust standard errors, or by summarizing outcomes at the cluster level). In terms of statistical approaches, the most commonly used analyses of entomological outcomes were negative binomial (NB) regression models (for count data, e.g. mosquito density) and their extensions (including mixed-effects and zero-inflated NB regression) (11/25, 44%), followed by Poisson regression, and generalized estimating equations (GEE). Simpler statistical methods for analysis of entomology outcomes, such as t-tests, ANOVA F-tests, or bootstrap analyses based on cluster-level summaries, were used less frequently.

**Fig. 7:**
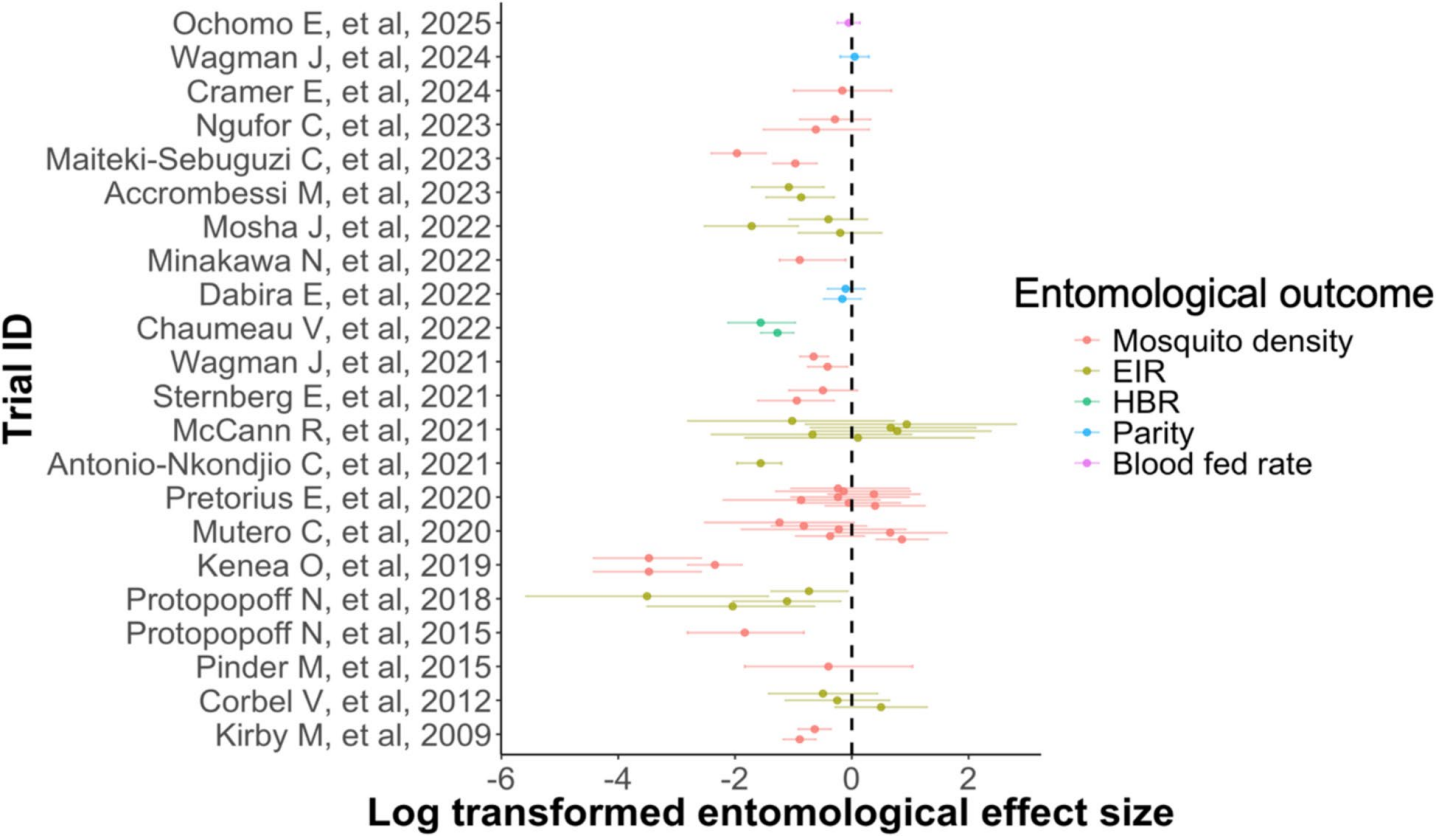
Distribution of log-transformed entomological effect sizes and 95% confidence intervals in 22 vector control cRCTs. Negative effect sizes indicate a reduction in outcomes, suggesting a benefit of the intervention, whereas positive values reflect increases in outcomes

Over 85% of studies reported entomological outcomes at the species complex or group level rather than distinguishing sibling species. Most trials presented data for the female *Anopheles* group as a whole, while others reported data for the *Anopheles gambiae* or *Anopheles funestus* group without further differentiation. The reported measures of entomological effects were odds ratios (5/25), risk ratios (7/25), density ratios (5/25), and incidence rate ratios (5/25). We excluded three trials that expressed entomological effects in terms of protective efficacy, risk difference and percentage change, because these measures were not directly comparable with the ratio-based effect measures used in the majority of trials. Visual inspection of the entomological effect sizes across trials shows high variability, both in the magnitude of the estimates and in the width of their confidence intervals (Fig. 7).

**Fig. 8:**
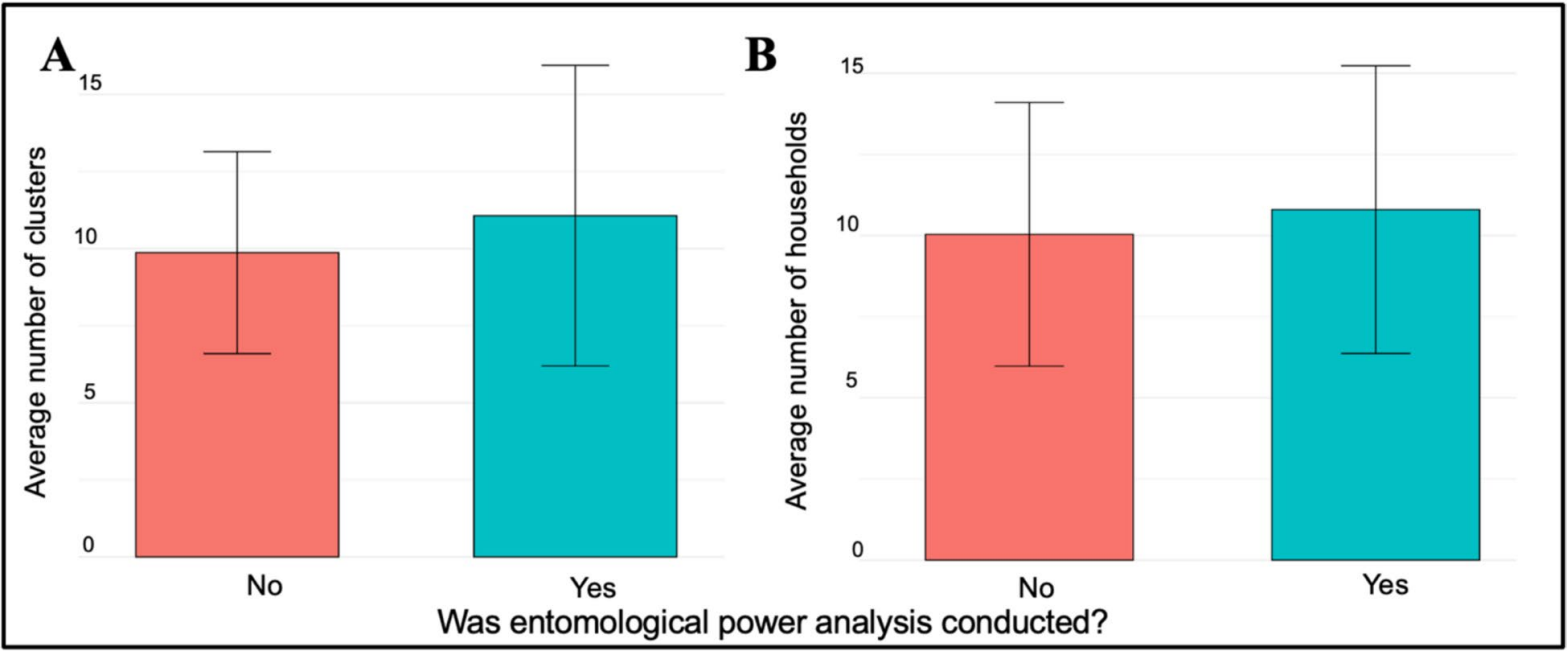
Association between entomological power analysis and **A** number of entomological clusters **B** number of entomological sampling points (households) in cRCTs

Of the three study design features, considered, the only significant predictor of entomological outcome precision was the number of clusters within the trial (positive association, β = 0.04552, p = 0.033). However, this should not be interpreted as indication that use of power analysis and the number of household sampling points within a cluster are unimportant for precision, as these factors are not independent. We noted that cRCTs that used power analysis to guide entomological sampling tended to collect data in a slightly (but non-significantly) higher number of clusters and sampling points (households) than those that did not (Fig. 8), with the choice to sample at more clusters likely being guided by power analysis in addition to other factors such as budget and personnel.

## Discussion

This review and meta-analysis systematically evaluated cRCTs of malaria vector control interventions, with a specific focus on how entomological outcomes are measured, the methodological rigor of the design features used to assess them, and the factors influencing the precision of entomological effect sizes. In contrast to generally robust and standardized approaches used to measure epidemiological outcomes, we found substantial variability in both the choice and methods used to measure entomological outcomes across vector control trials, and in the frequency and duration over which entomological data is collected. These issues may not necessarily represent weaknesses if the sampling methodologies, measurement period and frequency are appropriate for the intended intervention impact, however they do make it more difficult to assess and compare entomological impacts across cRCTs. A greater concern was the frequent and widespread evidence of weaknesses in the design features used to measure entomological outcomes. Most notable was the limited use of power analysis to guide entomological sampling efforts and non-random selection of household sampling points. The number and selection of clusters for entomological data collection was generally based on power analysis carried out for epidemiological outcomes, with entomology being carried out in all or a subset of epidemiology clusters, rather than being planned specifically for entomological outcomes. While the selection of clusters for entomological monitoring was often random, there were several trials where these were selected based on non-random factors such as easy access, high mosquito abundance or budget constraints example [50, 55, 59, 66, 69, 70]. Furthermore, the selection of entomological sampling points within clusters was commonly based on non or semi-random selection (random selection of the first household), or undefined. Through meta-analysis, we found that the precision with which entomological outcomes are measured is highly variable between trials; with the confidence intervals around estimated effect sizes narrowing as the number of clusters in which entomology data is measured increases. In combination, these results highlight that the rigor of design features for entomological measurements is considerably lower than for epidemiological outcomes in vector control cRCTs.

Our review revealed that the number of vector control cRCTs has been increasing since 1992, with a notable rise over the past decade. The majority of these trials were conducted in Africa, with East African countries such as Kenya, Tanzania, Uganda, and Ethiopia accounting for a disproportionate share, a pattern also noted by [71–76]. This regional concentration likely reflects the presence of high malaria transmission [77]. Our focus on English-language publications may have limited the inclusion of some studies from high-transmission settings, particularly in West Africa, where substantial research capacity exists but findings may be published in other languages, alongside the broader logistical and financial demands associated with conducting large-scale cRCTs, and historical legacies of where research institutes were established. However, large portions of North, Central, and Southern Africa remain underrepresented in current vector control trials. This limited geographical spread may undermine the generalizability of findings and restrict localized evidence generation. Although the WHO Vector Control Advisory Group (VCAG) recommended evaluating interventions in at least two different eco-epidemiological settings [78], our findings suggest that these settings are often drawn from a narrow subset of African countries. As a result, we may not be fully capturing the diversity of vector ecology, transmission dynamics, and programmatic contexts needed to assess intervention impact across the continent.

The range of main entomological outcomes and sampling methods used across trials was highly variable, reflecting both the biological diversity of malaria vectors and operational constraints within study settings. While some studies prioritized vector density indicators, others focused on transmission markers like the entomological inoculation rate (EIR), mosquito sporozoite rate, or parity. This variability may stem from differences in trial objectives, permissibility of different sampling approaches (e.g. Human Landing Catch), local vector behaviour, and intervention type. This pattern is also reflected at national surveillance level [14, 16] where they reported that the choice of entomological indicators varies across countries depending on the dominant vector species. However, while the choice of different entomological outcomes may be warranted, it also presents challenges for interpreting and comparing results across studies. These findings are consistent with previous reviews with a focus on understanding the value of entomological endpoints for assessing vector control interventions [15] and on improving the quality of vector control trials [9], which also noted the lack of standardisation in entomological outcomes. Our findings are also aligned with recent studies [14, 16, 79, 80] which collectively reported substantial heterogeneity in how entomological indicators are defined and measured across WHO regions [14, 16, 79, 80]. These analyses have revealed wide variation in the choice of mosquito sampling methods in surveillance, encompassing CDC light traps, pyrethrum spray catches, window exit traps, and human landing catches; with variation linked to prioritization of different unique strengths, limitations, and ethical considerations. Together, these findings highlight the need for clearer guidance on the selection and reporting of entomological outcomes, as well as greater harmonization of sampling approaches where feasible. There is substantial variability in the entomological outcomes used both across different intervention types and within trials evaluating the same intervention seen for ITN and endectocides where three and more outcomes were used. As we outlined in the introduction, standardization of sampling methods across all trials may not always be appropriate due to differences in vector species behaviour and ecology. However greater consistency in outcome definitions and, where possible, in sampling approaches for similar vector species and intervention contexts could improve comparability, reproducibility, and the overall utility of entomological evidence in vector control trials.

Recent research indicates that many vector control cRCTs are underpowered [9, 15], limiting their ability to detect targeted epidemiological effect sizes. This is often reflected by inconclusive results that may stem from design shortcomings rather than a true lack of efficacy. Our analysis of entomological study designs based on the ESDR framework showed there is considerable heterogeneity in the design and methodological rigour of entomological components in cRCTs. While common weaknesses were observed across trials, there is some indication of improvement over time, a trend that could be further strengthened through adequate funding and greater emphasis on the design and implementation of robust entomological monitoring. The number of clusters defined for entomological assessment was generally based on the power analysis conducted for epidemiological outcomes, with entomology conducted either in all or a subset of the clusters required for epidemiological measurements. While selecting entomological sampling sites from within epidemiological clusters is not inherently problematic, this approach becomes limiting when the number of clusters is not informed by power calculations specific to entomological outcomes, potentially resulting in insufficient power to detect prespecified effect sizes. Only a few trials conducted separate power analyses to define the number of entomology clusters required to detect pre-defined effect sizes of interest e.g. [27, 47, 48, 68, 81, 82]. Only half of these accounted for clustering effects. Such omissions can compromise the validity and interpretability of results.

Another area of concern in entomological design features was inconsistency in how sampling points (typically households) were selected. While randomisation at the cluster level was commonly inherited from epidemiological designs, household-level sampling often depended on convenience or logistical feasibility rather than methodological rigour. These findings are consistent with a previous review by Wilson et al. [9], who highlighted similar design and reporting deficiencies across vector-control trials. The recent studies reporting that epidemiological sampling designs in vector control cRCTs are often underpowered [71, 83] identified several key factors that lead to this. First, even when power analyses are performed, between-cluster heterogeneity (often expressed as the coefficient of variation k or intra-cluster correlation coefficient ICC) is commonly underestimated due to limited empirical data, leading to reduced true power in practice [71, 83]. Second, design assumptions are often not accurate or assumed effect sizes are too large, particularly when power calculations are based on optimistic expectations rather than on the smallest biologically or operationally meaningful effect that the study should be able to detect [84]. Third, unexpected transmission changes during the trial caused by weather anomalies, concurrent vector-control activities, or population behaviour shifts can markedly reduce observed event counts. These challenges that impact power for epidemiological assessment in cRCTs are likely similar and even more consequential for entomological outcomes, given that power analyses for entomological sampling were rarely conducted, and even when reported, may have relied on similarly inaccurate assumptions. Entomological systems are also highly variable and sensitive to environmental fluctuations, making them especially vulnerable to the same sources of misspecification and unexpected changes that undermine power in epidemiological outcomes. These parallels suggest that low power in entomological measurements may not only be common but structurally embedded, reinforcing the need for more robust, empirically informed, and adequately resourced entomological study designs. A fundamental challenge in entomological evaluations of vector control interventions is the difficulty of accurately sampling the relevant portion of the mosquito population affected by an intervention, as commonly used mosquito sampling tools capture only subsets of the vector population and may be influenced by vector behaviour and ecological context. While strengthening methodological rigor in trial design remains important, addressing these broader measurement challenges will also require continued methodological innovation and investment in improved tools for sampling and characterizing vector populations.

The observed association between the precision with which entomological outcomes were estimated and the number of clusters in which entomology surveillance was conducted is in line with expectation (precision increases number of clusters) and also suggests a potential benefit of formal sample size calculations in entomological trials. Although the LMM did not identify a significant association between reporting a power analysis for entomological outcomes and the precision of estimated entomological effect sizes, this null finding should be interpreted with caution. Our analysis included only 25 trials, which may have limited statistical power to detect modest associations. Trials that reported using power analysis for entomological measurements tended to include more clusters than those that did not, but these differences were small and not statistically significant. As such, while power analysis may plausibly influence study design choices, our results do not provide strong evidence for an effect on estimation of precision, and larger datasets would be required to robustly assess these relationships.

One concern that entomologists and trialists may have is that entomological outcomes may be more complex and heterogeneous than epidemiological ones. Entomological outcomes are subject to substantial spatial and temporal variability, requiring extensive sampling across clusters and time points to generate precise estimates, yet epidemiological outcomes carry greater weight in policy decisions. This could be construed as a disincentive for researchers to invest in improving the quality of entomological outcome measurement in trials as we propose here. However, we argue that acknowledging and attempting to improve at least some common weaknesses in entomological study designs will not incur significant cost and could conversely significantly increase the informativeness of trials. For example, our recommendations of improving the reporting of entomological outcomes would be cost neutral. Other recommendations such as performing power analysis would also not incur significant cost if it were done primarily to set reasonable expectation of what the trial can measure. Specifically, even when funds are insufficient to conduct entomological sampling in a large number of clusters/households that a power analysis may indicate is necessary to measure small-to-moderate impacts, performing such an analysis is still useful for assessment and reporting of the maximum effect size that the study design could detect. This knowledge could avoid premature dismissal of some interventions as having ‘no entomological impact’ when in fact the trial could only have detected very large ones. Also, the recommendation of random rather than haphazard selection of entomological sampling points (households) may reduce potential for bias but have little additional cost implications if it does not increase the total number of households or entomological surveys required. Finally, even when not powered as primary hypothesis-testing endpoints, entomological outcomes can provide important mechanistic and contextual insights that help explain how and why interventions succeed or fail. For example, entomological measurements can help determine whether interventions reduce mosquito density, whether reductions are associated with intervention coverage, whether species composition shifts over time, whether mosquito biting behaviour changes (e.g., shifts from indoor to outdoor biting), or whether factors such as emerging insecticide resistance may help explain negative epidemiological results.

## Conclusion

Entomological outcomes remain central to understanding how malaria vector-control interventions achieve their effects, even though they are often secondary endpoints. Our findings suggest that there is high variability in how entomological outcomes are measured in cRCTs, and consistent limitations in the study designs used to measure them including lack of power analysis and non-random selection of sampling points. The impact of these deficiencies on results interpretation is unclear, but they could provide one explanation for the often noted [85] inconsistent associations observed between entomological and epidemiological outcomes. Strengthening how entomological outcome measurements are selected, collected, and analysed is therefore essential to improving their interpretability and value for public health decision-making. Funders should recognize that investing in well-powered, well-reported entomological components is not an optional expense but a critical element for understanding intervention mechanisms and ensuring the success of large-scale malaria control trials. Likewise, integrating minimum entomological reporting standards into existing frameworks such as CONSORT and WHO guidance would help ensure more consistent, transparent, and policy-relevant evidence from future vector-control cRCTs.

## Supplementary Information

Supplementary material 1.

Supplementary material 2.

## Data Availability

All data extracted for this review were obtained from previously published studies, which are fully cited in the manuscript and have been deposited in the Dryad Digital Repository (DOI: 10.5061/dryad.z612jm6s9).

## Abbreviations

cRCTs: Cluster randomised controlled trials
IRS: Indoor residual spraying
ITN: Insecticide treated nets
LSM: Larvae source management
SE: Spatial emanator
HM: House modification
ITM: Insecticide treated material
ATB: Attractive targeted bait
ORS: Outdoor residual spraying
HBR: Human biting rate
EIR: Entomological inoculation rate
IR: Insecticide resistance
HLC: Human landing catch
CDC LT: Centre for disease control light trap
PSC: Pyrethrum spray catches
CBT: Cattle baited trap
PA: Prockopack aspirator
SSC: Space spray catches
LMM: Generalized linear mixed-effects model
