# Supplementary material for: A systematic review of entomological outcomes and sampling approaches used in the evaluation of cluster randomised controlled trials for malaria vector control products
Source: Malar J. 2026 Mar 22;25:187. doi: 10.1186/s12936-026-05866-4 (PMC13130592; doi:10.1186/s12936-026-05866-4)
Supplement: Supplementary file 1 — Supplementary material 1. [file 12936_2026_5866_MOESM1_ESM.docx]

**Supplementary file 1:** Search Strategy and search results of malaria vector control cRCTs

The subject heads for this systematic review are cluster randomised trials, malaria and vector control interventions

**Strategies for the subject heads (approach)**

*Randomised controlled trials:*

Randomised controlled trials OR randomised trials

*Cluster design-related terms:*

Cluster OR community intervention OR group intervention

*Malaria:*

Malaria OR mosquito OR anopheles OR plasmodium falciparum OR plasmodium vivax

*Vector control interventions:*

Vector control OR

insecticide treated nets OR Pyrethroid OR piperonyl butoxide OR PBO OR net OR

indoor residual spraying OR

spatial repellent OR Transfluthrin passive emanator OR

larvae source control OR LSM OR larvicide OR larval control OR environmental management OR attractive targeted bates OR Attractive Targeted Sugar Bait OR ATSB OR

Housing Modification OR

mosquito control OR Peridomestic OR

repel and lure devices OR Sterile insect technique OR SIT

We will search the following databases for relevant trials.

- MEDLINE (Ovid),
- Embase (Ovid),
- Web of Science Core Collection,
- Global Index Medicus.

We will also search the following trial registries.

- The World Health Organization International Clinical Trials Registry Platform [www.who.int/trialsearch](http://www.who.int/trialsearch)
- ClinicalTrials.gov [www.clinicaltrials.gov](http://www.clinicaltrials.gov).
- CENTRAL (The Cochrane Central Register of Controlled Trials)

**MEDLINE (ovid):**

**Randomised controlled trials**

1. “randomised controlled trial".pt.
2. animals/
3. humans/
4. 2 NOT (2 AND 3)
5. 1 NOT 4

**Cluster design related terms**

1. cluster$ adj2 randomi$.tw.
2. ((communit$ adj2 intervention$) OR (communit$ adj2 randomi$)).tw.
3. group$ randomi$.tw.
4. 6 OR 7 OR 8
5. intervention?.tw.
6. cluster analysis/
7. health promotion/
8. program evaluation/
9. health education/
10. 10 OR 11 OR 12 OR 13 OR 14
11. 9 OR 15

**Malaria related words**

1. Malaria/
2. (malaria).tw
3. Anopheles/
4. (anopheles or mosquito*).tw
5. or/1-4 [mosquitoes/malaria concept]

**Vector control interventions**

1. (indoor residual spraying or irs).tw
2. (house spray*).tw
3. Insecticides/
4. Pyrethrins/
5. (malathion or fenitrothion or pirimiphos‐methyl or bendiocarb or propoxur or alpha‐cypermethrin or bifenthrin or cyfluthrin or deltamethrin or etofenprox or lambda‐cyhalothrin or DDT).tw
6. Insecticide‐Treated Bednet$/
7. mosquito control/
8. (insecticide‐treated bednet$ OR insecticide‐treated net$ OR Long‐lasting insecticidal net$ OR LLIN$ OR ITN$ OR LN$ OR bed net$ or long‐lasting net$).tw
9. (spatial repellent$ OR insect repellent$ OR Transfluthrin OR emanator$).tw
10. ((Peridomestic AND repel AND lure device$).tw
11. (Sterile insect technique$ OR SIT).tw
12. (attractive targeted bate$ OR Attractive Targeted Sugar Bait$ OR ATSB).tw
13. (environmental management$ OR Housing Modification$).tw
14. (larva$ source control OR larva$ source management OR larva$ control OR larvicide$).tw
15. (house management OR housing improvement$ OR house modification OR source reduction$).tw
16. or/22-36 [intervention/control concept]
17. 5 AND 16 AND 21 AND 37

“$” allows for truncation of words so that variations such as “randomization”, “randomisation”, “randomized” are included; adj refers to the adjacency operator to accommodate terms such as “community-based randomized trial"; pt refers to publication type; ? refers to optional wildcard character retrieving 1 or 0 characters;/refers to MeSH; tw refers to text words in the title and abstract.

**Results:**

**Database:
Ovid MEDLINE(R) ALL <1946 to July 19, 2024>**

| **#** | **Query** | **Results from 16 Aug 2024** |
| --- | --- | --- |
| 1 | randomized controlled trial.pt. | 619,082 |
| 2 | animals.hw. | 7,489,473 |
| 3 | humans.hw. | 22,161,022 |
| 4 | 2 not (2 and 3) | 5,215,236 |
| 5 | 1 not 4 | 607,464 |
| 6 | (cluster$ adj2 randomi$).tw. | 19,515 |
| 7 | cluster analysis.sh. | 71,209 |
| 8 | health promotion.sh. | 83,425 |
| 9 | program evaluation.sh. | 68,005 |
| 10 | health education.sh. | 64,441 |
| 11 | 6 or 7 or 8 or 9 or 10 | 282,918 |
| 12 | Malaria.sh. | 52,881 |
| 13 | malaria.tw. | 94,715 |
| 14 | Anopheles.sh. | 15,804 |
| 15 | (anopheles or mosquito*).tw. | 58,737 |
| 16 | 12 or 13 or 14 or 15 | 148,796 |
| 17 | entomological.tw. | 4,921 |
| 18 | epidemiological.tw. | 225,889 |
| 19 | (indoor residual spraying or IRS).tw. | 13,602 |
| 20 | house spray*.tw. | 212 |
| 21 | Insecticides.sh. | 53,151 |
| 22 | Pyrethrins.sh. | 10,197 |
| 23 | (malathion or fenitrothion or pirimiphos methyl or bendiocarb or propoxur or alpha cypermethrin or bifenthrin or cyfluthrin or deltamethrin or etofenprox or lambda cyhalothrin or DDT).tw. | 22,758 |
| 24 | Insecticide Treated Bednet$.sh. | 1,630 |
| 25 | mosquito control.sh. | 10,921 |
| 26 | (insecticide treated bednet$ or insecticide treated net$ or Long lasting insecticidal net$ or LLIN$ or ITN$ or LN$ or bed net$ or long lasting net$).tw. | 115,224 |
| 27 | (spatial repellent$ or insect repellent$ or Transfluthrin or emanator$).tw. | 1,414 |
| 28 | (Peridomestic or repel or lure device$).tw. | 2,829 |
| 29 | (Sterile insect technique$ or SIT).tw. | 16,186 |
| 30 | (attractive targeted bate$ or Attractive Targeted Sugar Bait$ or ATSB).tw. | 104 |
| 31 | (environmental management$ or Housing Modification$).tw. | 3,689 |
| 32 | (larva$ source control or larva$ source management or larva$ control or larvicide$).tw. | 2,004 |
| 33 | (house management or housing improvement$ or house modification or source reduction$).tw. | 638 |
| 34 | 17 or 18 or 19 or 20 or 21 or 22 or 23 or 24 or 25 or 26 or 27 or 28 or 29 or 30 or 31 or 32 or 33 | 452,215 |
| **35** | **5 and 11 and 16 and 34** | **169** |

**Database:
Embase 1947-Present, updated daily**

| **#** | **Query** | **Results from 16 Aug 2024** |
| --- | --- | --- |
| 1 | randomized controlled trial.tw. | 151,440 |
| 2 | animal.sh. | 2,174,896 |
| 3 | human.sh. | 28,196,700 |
| 4 | 2 not (2 and 3) | 1,645,853 |
| 5 | 1 not 4 | 151,287 |
| 6 | (cluster$ adj2 randomi$).tw. | 23,574 |
| 7 | ((communit$ adj2 intervention$) or (communit$ adj2 randomi$)).tw. | 14,690 |
| 8 | group$ randomi$.tw. | 6,595 |
| 9 | 6 or 7 or 8 | 43,732 |
| 10 | intervention?.tw. | 1,920,579 |
| 11 | cluster analysis.sh. | 72,378 |
| 12 | health promotion.sh. | 116,110 |
| 13 | program evaluation.sh. | 19,976 |
| 14 | health education.sh. | 120,890 |
| 15 | 10 or 11 or 12 or 13 or 14 | 2,173,452 |
| 16 | 9 or 15 | 2,182,139 |
| 17 | Malaria.sh. | 98,741 |
| 18 | malaria.tw. | 118,734 |
| 19 | Anopheles.sh. | 13,079 |
| 20 | (anopheles or mosquito*).tw. | 69,603 |
| 21 | 17 or 18 or 19 or 20 | 194,356 |
| 22 | entomological.tw. | 11,020 |
| 23 | epidemiological.tw. | 303,693 |
| 24 | (indoor residual spraying or IRS).tw. | 17,906 |
| 25 | house spray*.tw. | 281 |
| 26 | Insecticides.sh. | 2 |
| 27 | Pyrethrins.tw. | 557 |
| 28 | (malathion or fenitrothion or pirimiphos methyl or bendiocarb or propoxur or alpha cypermethrin or bifenthrin or cyfluthrin or deltamethrin or etofenprox or lambda cyhalothrin or DDT).tw. | 29,110 |
| 29 | Insecticide Treated Bednet$.sh. | 4 |
| 30 | mosquito control.sh. | 2,404 |
| 31 | (insecticide treated bednet$ or insecticide treated net$ or Long lasting insecticidal net$ or LLIN$ or ITN$ or LN$ or bed net$ or long lasting net$).tw. | 159,121 |
| 32 | (spatial repellent$ or insect repellent$ or Transfluthrin or emanator$).tw. | 1,716 |
| 33 | (Peridomestic or repel or lure device$).tw. | 2,951 |
| 34 | (Sterile insect technique$ or SIT).tw. | 23,570 |
| 35 | (attractive targeted bate$ or Attractive Targeted Sugar Bait$ or ATSB).tw. | 102 |
| 36 | (environmental management$ or Housing Modification$).tw. | 4,556 |
| 37 | (larva$ source control or larva$ source management or larva$ control or larvicide$).tw. | 2,450 |
| 38 | (house management or housing improvement$ or house modification or source reduction$).tw. | 847 |
| 39 | 22 or 23 or 24 or 25 or 26 or 27 or 28 or 29 or 30 or 31 or 32 or 33 or 34 or 35 or 36 or 37 or 38 | 550,656 |
| **40** | **5 and 16 and 21 and 39** | **177** |

**Web of science Core Collection: 1900-2024**

| # | Search Query | Database | Results |
| --- | --- | --- | --- |
| 1 | TS=(randomised controlled trial) and Preprint Citation Index (Exclude – Database) | All Databases | 923998 |
| 2 | TS=(animal) and Preprint Citation Index (Exclude – Database) | All Databases | 33022244 |
| 3 | TS=(human) and Preprint Citation Index (Exclude – Database) | All Databases | 41941000 |
| 4 | #2 NOT (#2 AND #3) and Preprint Citation Index (Exclude – Database) | All Databases | 12198048 |
| 5 | #1 NOT #4 and Preprint Citation Index (Exclude – Database) | All Databases | 913003 |
| 6 | (TI=((communit? NEAR/2 intervention? ) OR (communit? NEAR/2 randomi? )) OR AB=((communit? NEAR/2 intervention? ) OR (communit? NEAR/2 randomi? ))) and Preprint Citation Index (Exclude – Database) | All Databases | 12403 |
| 7 | TI=(group? randomi?) OR AB=(group? randomi?) and Preprint Citation Index (Exclude – Database) | All Databases | 35 |
| 8 | TI=(cluster? NEAR/2 randomi?) OR AB=(cluster? NEAR/2 randomi?) and Preprint Citation Index (Exclude – Database) | All Databases | 2 |
| 9 | #6 OR #7 OR #8 and Preprint Citation Index (Exclude – Database) | All Databases | 12439 |
| 10 | (TI=intervention$ OR AB=intervention$) and Preprint Citation Index (Exclude – Database) | All Databases | 2632520 |
| 11 | TS=("cluster analysis" ) and Preprint Citation Index (Exclude – Database) | All Databases | 238582 |
| 12 | TS=("health promotion" ) and Preprint Citation Index (Exclude – Database) | All Databases | 192858 |
| 13 | TS=("program evaluation" ) and Preprint Citation Index (Exclude – Database) | All Databases | 94781 |
| 14 | TS=("health education" ) and Preprint Citation Index (Exclude – Database) | All Databases | 212327 |
| 15 | #10 OR #11 OR #12 OR #13 OR #14 and Preprint Citation Index (Exclude – Database) | All Databases | 3215018 |
| 16 | #15 OR #9 and Preprint Citation Index (Exclude – Database) | All Databases | 3215047 |
| 17 | TS=(Malaria ) and Preprint Citation Index (Exclude – Database) | All Databases | 276511 |
| 18 | TS=(Anopheles ) and Preprint Citation Index (Exclude – Database) | All Databases | 71046 |
| 19 | (TI=(malaria ) OR AB=(malaria )) and Preprint Citation Index (Exclude – Database) | All Databases | 216671 |
| 20 | (TI=(anopheles OR mosquito* ) OR AB=(anopheles OR mosquito* )) and Preprint Citation Index (Exclude – Database) | All Databases | 228527 |
| 21 | #17 OR #18 OR #19 OR #20 and Preprint Citation Index (Exclude – Database) | All Databases | 461419 |
| 22 | (TI=(indoor residual spraying or IRS ) OR AB=(indoor residual spraying or IRS )) and Preprint Citation Index (Exclude – Database) | All Databases | 37915 |
| 23 | (TI=(house spray? ) OR AB=(house spray? )) and Preprint Citation Index (Exclude – Database) | All Databases | 4778 |
| 24 | TS=(Insecticide?) and Preprint Citation Index (Exclude – Database) | All Databases | 295906 |
| 25 | TS=(Pyrethrin?) and Preprint Citation Index (Exclude – Database) | All Databases | 16844 |
| 26 | TI=(malathion or fenitrothion or pirimiphos‐methyl or bendiocarb or propoxur or alpha‐cypermethrin or bifenthrin or cyfluthrin or deltamethrin or etofenprox or lambda‐cyhalothrin or DDT) OR AB=(malathion or fenitrothion or pirimiphos‐methyl or bendiocarb or propoxur or alpha‐cypermethrin or bifenthrin or cyfluthrin or deltamethrin or etofenprox or lambda‐cyhalothrin or DDT) and Preprint Citation Index (Exclude – Database) | All Databases | 101225 |
| 27 | TS=(Insecticide‐Treated Bednet) and Preprint Citation Index (Exclude – Database) | All Databases | 479 |
| 28 | TS=(mosquito control) and Preprint Citation Index (Exclude – Database) | All Databases | 109183 |
| 29 | TI=(insecticide‐treated bednet* or insecticide‐treated net* or Long‐lasting insecticidal net* or LLIN* or bed net* or long‐lasting net*) OR AB=(insecticide‐treated bednet* or insecticide‐treated net* or Long‐lasting insecticidal net* or LLIN* or ITN* or LN* or bed net* or long‐lasting net*) and Preprint Citation Index (Exclude – Database) | All Databases | 412392 |
| 30 | TI=(spatial repellent$ OR insect repellent$ OR Transfluthrin OR emanator$) OR AB=(spatial repellent$ OR insect repellent$ OR Transfluthrin OR emanator$) and Preprint Citation Index (Exclude – Database) | All Databases | 20284 |
| 31 | TI=(Peridomestic or repel or lure device$) OR AB=(Peridomestic or repel or lure device$) and Preprint Citation Index (Exclude – Database) | All Databases | 99293 |
| 32 | TI=(Sterile insect technique$ OR SIT) OR AB=(Sterile insect technique$ OR SIT) and Preprint Citation Index (Exclude – Database) | All Databases | 272064 |
| 33 | TI=(attractive targeted bate$ OR Attractive Targeted Sugar Bait$ OR ATSB) OR AB=(attractive targeted bate$ OR Attractive Targeted Sugar Bait$ OR ATSB) and Preprint Citation Index (Exclude – Database) | All Databases | 276 |
| 34 | TI=(environmental management$ OR Housing Modification$) OR AB=(environmental management$ OR Housing Modification$) and Preprint Citation Index (Exclude – Database) | All Databases | 358334 |
| 35 | TI=(larva$ source control OR larva$ source management OR larva$ control OR larvicide$) OR AB=(larva$ source control OR larva$ source management OR larva$ control OR larvicide$) and Preprint Citation Index (Exclude – Database) | All Databases | 146978 |
| 36 | TI=(house management OR housing improvement$ OR house modification OR source reduction$) OR AB=(house management OR housing improvement$ OR house modification OR source reduction$) and Preprint Citation Index (Exclude – Database) | All Databases | 406496 |
| 37 | TI=(entomological or epidemiological) OR AB=(entomological or epidemiological) and Preprint Citation Index (Exclude – Database) | All Databases | 425808 |
| 38 | #37 OR #36 OR #35 OR #34 OR #33 OR #32 OR #31 OR #30 OR #29 OR #28 OR #27 OR #26 OR #25 OR #24 OR #23 OR #22 and Preprint Citation Index (Exclude – Database) | All Databases | 2482699 |
| 39 | #38 AND #21 AND #16 AND #5 and Preprint Citation Index (Exclude – Database) | All Databases | 802 |
| 40 | #38 AND #21 AND #16 AND #5 and Preprint Citation Index (Exclude – Database) and English (Languages) | All Databases | 802 |
| 41 | #38 AND #21 AND #16 AND #5 and Preprint Citation Index (Exclude – Database) and English (Languages) and Web of Science Core Collection (Database) | All Databases | 559 |

**Global Index Medicus**

TW:((randomised controlled trial) OR (communit? NEAR/2 intervention? ) OR (communit? NEAR/2 randomi?) OR (group? randomi?) OR (cluster? NEAR/2 randomi?) OR intervention$ OR (cluster analysis) OR (health promotion) OR (program evaluation) OR (health education))

AND

TW:(Malaria OR anopheles OR mosquito*)

AND

TW:(entomological OR epidemiological OR (indoor residual spraying) or IRS OR (house spray?) OR Insecticide? OR Pyrethrin? OR malathion or fenitrothion or (pirimiphos methyl) or bendiocarb or propoxur or (alpha cypermethrin) or bifenthrin or cyfluthrin or deltamethrin or etofenprox or (lambda cyhalothrin) or DDT OR (insecticide treated bednet*) or (insecticide treated net*) or (Long lasting insecticidal net*) or LLIN* or (bed net*) or (long lasting net*) OR

(spatial repellent$) OR (insect repellent$) OR Transfluthrin OR emanator$ OR Peridomestic or repel or lure device$ OR (Sterile insect technique$) OR SIT OR (attractive targeted bate$) OR (Attractive Targeted Sugar Bait$) OR ATSB OR (environmental management$) OR (Housing Modification$) OR (larva$ source control) OR (larva$ source management) OR (larva$ control) OR larvicide$ OR (house management) OR (housing improvement$) OR (house modification) OR (source reduction$))

((randomised controlled trial) OR (communit? near/2 intervention? ) OR (communit? near/2 randomi?) OR (group? randomi?) OR (cluster? near/2 randomi?) OR intervention* OR (cluster analysis) OR (health promotion) OR (program evaluation) OR (health education)) AND (malaria OR anopheles OR mosquito*) AND (entomological OR epidemiological OR (indoor residual spraying) OR irs OR (house spray?) OR insecticide? OR pyrethrin? OR malathion OR fenitrothion OR (pirimiphos methyl) OR bendiocarb OR propoxur OR (alpha cypermethrin) OR bifenthrin OR cyfluthrin OR deltamethrin OR etofenprox OR (lambda cyhalothrin) OR ddt OR (insecticide treated bednet*) OR (insecticide treated net*) OR (long lasting insecticidal net*) OR llin* OR (bed net*) OR (long lasting net*) OR (spatial repellent*) OR (insect repellent*) OR transfluthrin OR emanator* OR peridomestic OR repel OR lure device* OR (sterile insect technique*) OR sit OR (attractive targeted bate*) OR (attractive targeted sugar bait*) OR atsb OR (environmental management*) OR (housing modification*) OR (larva* source control) OR (larva* source management) OR (larva* control) OR larvicide* OR (house management) OR (housing improvement*) OR (house modification) OR (source reduction*)) AND ( la:("en"))

**TOTAL OF 290**

**CENTRAL**

Search Name:

Date Run: 16/08/2024 20:23:07

Comment:

ID Search Hits

#1 randomized controlled trial 1112827

#2 (animal$):ti,ab,kw 24183

#3 (human$):ti,ab,kw 811899

#4 #2 NOT (#2 AND #3) 8808

#5 #1 NOT #4 1108633

#6 group? randomi? 2111

#7 (communit$ intervention$ ) OR (communit$ randomi$) 91

#8 cluster? NEAR/2 randomi? 4

#9 (intervention$):ti,ab,kw 566302

#10 (cluster analysis) OR (health promotion) OR (program evaluation) OR (health education) 115077

#11 #6 OR #7 OR #8 OR #9 OR #10 609977

#12 malaria or anopheles 7906

#13 (anopheles or mosquito):ti,ab,kw 1270

#14 #12 or #13 8309

#15 ((indoor residual spraying) or IRS):ti,ab,kw 807

#16 (entomological):ti,ab,kw 303

#17 (epidemiological):ti,ab,kw 6877

#18 (house spray?):ti,ab,kw 83

#19 Insecticide? or Pyrethrin? 1327

#20 (malathion or fenitrothion or pirimiphos‐methyl or bendiocarb or propoxur or alpha‐cypermethrin or bifenthrin or cyfluthrin or deltamethrin or etofenprox or lambda‐cyhalothrin or DDT):ti,ab,kw 572

#21 ((insecticide‐treated bednet*) or (insecticide‐treated net*) or (Long‐lasting insecticidal net*) or LLIN* or (bed net*) or (long‐lasting net*)):ti,ab,kw 1346

#22 mosquito control 903

#23 ((spatial repellent$) OR (insect repellent$) OR Transfluthrin OR emanator$):ti,ab,kw 115

#24 (Peridomestic or repel or lure device$):ti,ab,kw 37

#25 (Sterile insect technique$ OR SIT):ti,ab,kw 6820

#26 ((attractive targeted bate$) OR (Attractive Targeted Sugar Bait$) OR ATSB):ti,ab,kw 8

#27 ((environmental management$) OR (Housing Modification$)):ti,ab,kw 1353

#28 ((larva$ source control) OR (larva$ source management) OR (larva$ control OR larvicide$)):ti,ab,kw 193

#29 ((house management) OR (housing improvement$) OR (house modification) OR (source reduction$)):ti,ab,kw 3115

#30 #15 OR #16 OR #17 OR #18 OR #19 OR #20 OR #21 or #22 OR #23 OR #24 OR #25 OR #26 or #27 OR #28 OR #29 21346

#31 #5 AND #11 AND #14 AND #30 **617 Trials**

Clinical trials.gov

Showing results for: **Malaria OR mosquito OR anopheles | Other terms: cluster randomized trials OR intervention OR randomized controlled trial OR communit$ intervention OR cluster analysis | vector control OR insecticide treated bednets OR mosquito control OR LLIN OR spatial repellent OR house management OR Sterile insect technique OR SIT OR environmental management OR LLIN OR ITN OR IRS OR house spray OR indoor residual spraying OR larva$ source management | Completed studies**

**Total of 63 studies**
