# Supplementary material for: A systematic review of entomological outcomes and sampling approaches used in the evaluation of cluster randomised controlled trials for malaria vector control products
Source: Malar J. 2026 Mar 22;25:187. doi: 10.1186/s12936-026-05866-4 (PMC13130592; doi:10.1186/s12936-026-05866-4)
Supplement: Supplementary file 2 — Supplementary material 2. [file 12936_2026_5866_MOESM2_ESM.docx]

**Supplementary file 2:**

**Entomological Study Design Risk (ESDR) Ranking:**

The ESDR evaluated four domains: D1 – whether a power analysis was conducted for entomological outcomes; D2 – whether clustering was accounted for in the entomological power analysis; D3 – whether clusters were randomly selected for entomological outcome measurement; and D4 – whether household sampling points were randomly selected for entomological outcome measurement. Each domain was ranked using a three-level scale: low risk (green), some concerns (yellow), and high risk (red).

For D1, trials that reported conducting a power analysis were judged low risk, while those that did not conduct or report were judged high risk. For D2, trials that conducted a power analysis and accounted for clustering were rated as low risk but those that did not state if clustering was accounted for were rated as having some concerns, whereas those that did not conduct a power analysis at all were rated high risk. For D3, trials that reported random selection of clusters were judged low risk, while those that did not were judged high risk. For D4, trials that reported random selection of sampling points were judged low risk, those with no randomisation were judged high risk, and those with semi-random selection (e.g. the first point chosen randomly with subsequent points chosen from the first) or unclear methods were judged as having some concerns.

**Risk of bias of entomological study design of the 25 trials in the meta-analysis**

**
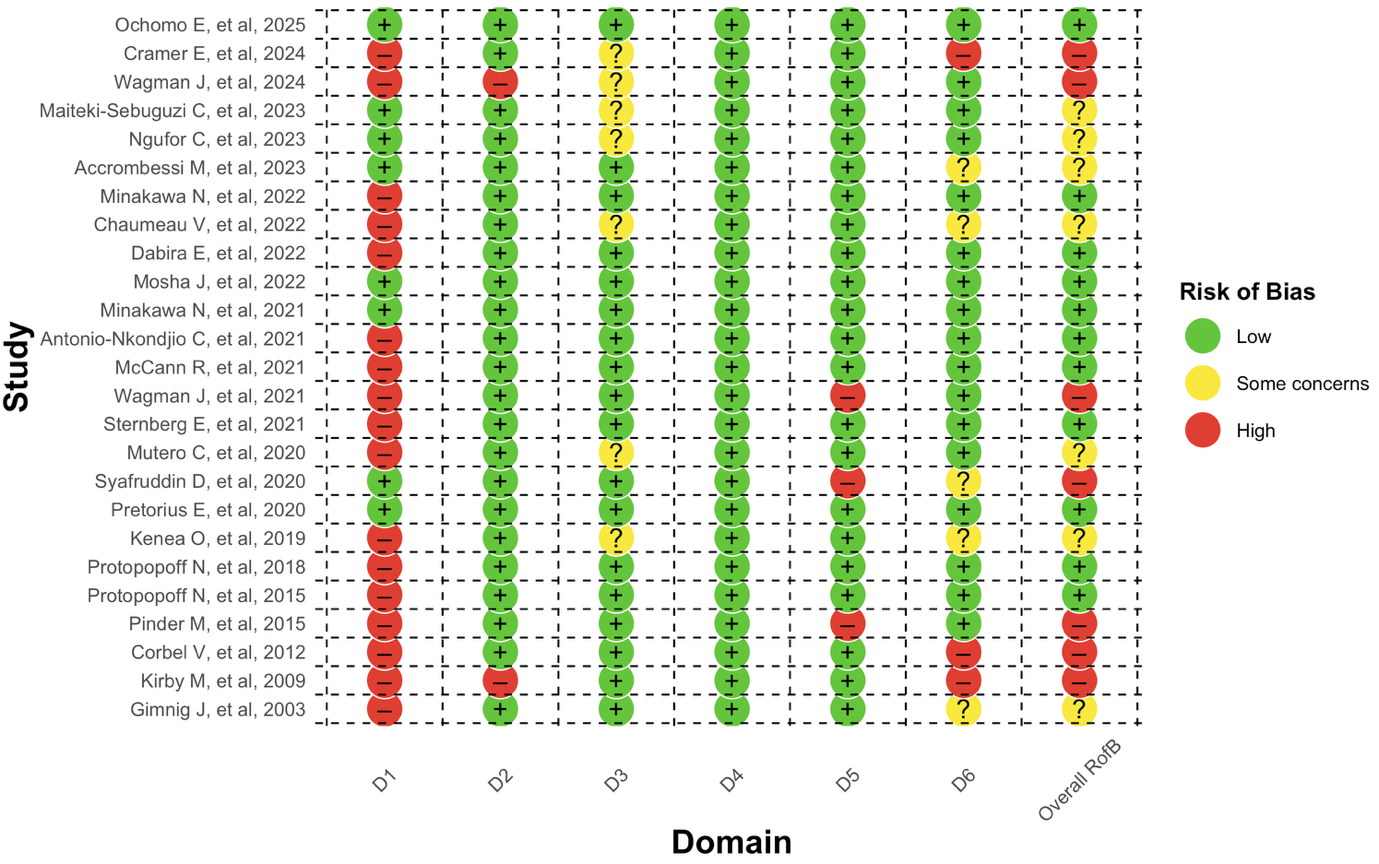
**

**Fig 1:** Results of the Cochrane risk of bias assessment are shown for the 25 trials included in the analysis of entomological effect size precision. The assessment was conducted using the standard Cochrane risk of bias framework [1], applying the same core domains used for randomized controlled trials, but with judgments made specifically in relation to entomological outcomes. The six domains assessed were: D1, bias due to unmasking of the intervention; D2, bias due to missing entomological outcome data; D3, bias due to awareness of intervention assignment during analysis; D4, completeness of reporting of planned results; D5, randomisation of clusters for entomological data collection; and D6, randomisation of households for entomological data collection.

A large proportion of trials were judged to be at high risk of bias for Domain 1 (bias due to unmasking of the intervention); however, this was not included in the overall judgement because masking participants in vector control trials is rarely feasible (often interventions are highly visible and cannot be hidden). In Domain 2 (bias due to missing entomological outcome data), only 2 trials were rated at high risk, reflecting challenges with incomplete data capture. Domain 3 (bias due to awareness of intervention assignment during analysis) showed mostly low risk, with a few studies rated as “some concerns” due to not reporting of this masking. Similarly, Domain 4 (completeness of reporting of planned results) was predominantly low risk. In contrast, Domains 5 and 6, which assessed the randomisation of clusters and households for entomological data collection, were also predominantly rated as low risk, with only a small number of trials showing elevated concern. In Domain 5, almost all trials were judged low risk, indicating that cluster randomisation procedures were consistently well applied. Domain 6 showed similarly strong performance for these trials included in the precision analysis rather than the overall 46 trials included in this review, though a few trials were rated as “some concerns” or “high risk” due to incomplete descriptions of how households were selected or the use of non or semi-random procedures. When synthesised into an overall risk-of-bias judgement, the majority of trials remained classified as low risk, with only a handful falling into the “some concerns” or “high risk” categories (Fig. 1). Overall, among the 25 trials included in the precision analysis, the Cochrane risk of bias assessment suggested that explicit risks of study quality bias were generally low. It is important to note that this assessment was conducted on a subset of trials and is distinct from the broader ESDR-based assessment applied to all 46 included trials. The 25 trials contributing to the precision analysis were predominantly more recent studies (post-2009), which tended to show more complete reporting of entomological outcomes and study procedures. This likely contributed to the more favourable risk of bias profile observed in this subset and should not be interpreted as representative of all trials included in the review.

**Reference:**

1. Higgins, J.P., et al., *The Cochrane Collaboration's tool for assessing risk of bias in randomised trials.* BMJ, 2011. **343**: p. d5928.
